# Nanoparticle Saltwater Fish Powder and Cell Protein Pathways in Enamel Density Enhancement

**DOI:** 10.1055/s-0045-1813035

**Published:** 2025-12-23

**Authors:** Sandy Christiono, Islamy Rahma Hutami, Fourier Dzar Eljabbar Latief, Yayun Siti Rochmah, Grahita Aditya, Suparmi Suparmi, Shaista Afroz, Savira Nurazky Yuniar

**Affiliations:** 1 Department of Pediatric Dentistry, Faculty of Dentistry, Universitas Islam Sultan Agung, Semarang, Indonesia; 2 Department of Orthodontics, Faculty of Dentistry, Universitas Islam Sultan Agung, Semarang, Indonesia; 3 Physics of Earth and Complex Systems, Faculty of Mathematics and Natural Sciences, Bandung Institute of Technology, Bandung, Indonesia; 4 Department of Oral and Maxillofacial Surgery, Faculty of Dentistry, Universitas Islam Sultan Agung, Semarang, Indonesia; 5 Department of Biology, Faculty of Medicine, Universitas Islam Sultan Agung, Semarang, Indonesia; 6 Department of Prosthodontic/Dental Material, ZA Dental College, Aligarh Muslim University, Aligarh, India; 7 Faculty of Dentistry, Universitas Islam Sultan Agung, Semarang, Indonesia

**Keywords:** amelogenin, FABP-3, fetal mice, enamel density, nanoparticle, saltwater fish powder

## Abstract

**Objective:**

This study aims to evaluate the effect of nanoparticle saltwater fish powder on amelogenin expression and FABP-3 levels in fetal mice, with the goal of enhancing enamel density and supporting tooth mineralization. To date, this relationship has not been clearly explained in previous studies.

**Materials and Methods:**

This randomized experimental study involved 16 pregnant mice, divided into two groups: control and treatment. The treatment group received nanoparticle saltwater fish powder (2.14 mg/mL) orally three times daily, while the control group received distilled water. On gestational day 18, placental and fetal jaw tissues were collected. Amelogenin and FABP-3 expressions were analyzed by immunohistochemistry (IHC), while enamel density was assessed using micro-computed tomography (µCT).

**Statistical Analysis:**

Data were analyzed using SPSS 26 and presented as mean ± SD. Group differences were tested using an independent *t* -test; results were considered significant at *p* < 0.05.

**Results:**

IHC analysis revealed significantly increased amelogenin expression in the treatment group receiving nanoparticle saltwater fish powder (4.34 ± 3.26) compared to the control group (0.49 ± 0.40), with a *p* -value of 0.005. FABP-3 expression was also significantly higher in the treatment group (2.26 ± 0.85) than in the control (1.50 ± 0.40), with a *p* -value of 0.038. µCT imaging displayed differences in enamel density between the treatment group (228.73 ± 5.31) versus the control (220.75 ± 5.95), with a *p* -value of 0.022.

**Conclusion:**

Nanoparticle saltwater fish powder modulates amelogenin expression during enamel secretion and enhances FABP-3 expression, suggesting potential benefits for promoting enamel development through nutritional interventions. Moreover, µCT analysis revealed an increase in the mean enamel density.

## Introduction

Enamel, the outermost protective layer of teeth, serves as the first line of defense against mechanical and microbial insults. Its formation, known as amelogenesis, occurs predominantly during fetal development and is critical for determining the long-term resilience of dental tissue. 1 Enamel is particularly susceptible to caries and structural defects if its quality and density are compromised, emphasizing the importance of understanding factors that influence its development. 2 Amelogenesis is generally divided into five stages: the pre-ameloblast, pre-secretory, secretory, transition, and maturation phases. These stages are characterized by morphological and functional changes in ameloblasts, a monolayer of specialized epithelial cells responsible for enamel matrix secretion and subsequent mineralization. 3 During the maturation phase, significant mineral deposition occurs, leading to the removal of the organic matrix components and increased enamel hardness and density. 4 5

Amelogenin is a key protein involved in dental enamel formation 6 and plays a crucial role in regulating hydroxyapatite crystal growth and organization, thus directly affecting the enamel mineralization density and mechanical structure. 7 8 Fatty acid-binding proteins (FABPs), particularly FABP-3, are lipid transporters implicated in cellular lipid metabolism, which has been suggested to influence ameloblast function and enamel mineralization through modulation of lipid availability and signaling pathways. 9 Although the direct involvement of FABP-3 in enamel-forming cells has not yet been fully established, its known role in lipid handling highlights a potential contribution to enamel biomineralization, warranting further investigation into its specific function in ameloblasts.

Marine biota, especially saltwater fish, are rich sources of essential nutrients such as protein, minerals, and bioactive compounds including omega-3 fatty acids, calcium, vitamin A, D, and B12, sodium, fluoride, and selenium. 10 These nutrients are known to influence key pathways involved in enamel development. For instance, omega-3 fatty acids like eicosapentaenoic acid (EPA) and docosahexaenoic acid (DHA) support lipid metabolism and have been shown to promote FABP-3 expression, facilitating lipid metabolism and mineralization during amelogenesis. 11 Additionally, amino acids like histidine and glycine are fundamental constituents of amelogenin, influencing its synthesis and functionality. Vitamins and minerals such as vitamin D, calcium, and phosphorus are vital for hydroxyapatite crystal formation. 12 13 14 However, the bioavailability of nutrients from natural dietary sources is often limited due to factors such as molecular and absorption insufficiencies, such as low solubility, limited intestinal absorption, and restricted placental transfer.

Recent advances in nanotechnology offer promising opportunities to enhance nutrient delivery. 15 Nanoparticle formulations, ranging from 50 to 300 nm in size, increase bioavailability by facilitating better absorption and cellular uptake of minerals and bioactive compounds such as calcium, phosphate, and omega-3 fatty acids. 16 Applying nanoparticle technology to nutrient delivery could potentiate the effects of marine-derived nutrients on amelogenesis, yet scientific evidence on their specific impact on fetal enamel formation remains limited.

Therefore, this study aims to investigate the effects of nanoparticle saltwater fish powder supplementation on the expression of key enamel proteins (amelogenin) and FABP-3, in fetal mice. FABP-3 was assessed in the placenta as an indicator of the ability of nanoparticles to cross the placental barrier. Furthermore, this research evaluates the influence of this intervention on enamel density using micro-computed tomography (micro-CT/μCT), contributing to the understanding of nutritional strategies for optimal fetal enamel development.

## Materials and Methods

### Study Design

This randomized experimental study was conducted using a single-blind design in which the animals were randomly allocated into control and treatment groups. The researchers administering the intervention were aware of group assignments, while the outcome evaluators remained blinded to reduce the risk of bias. The study was approved by the Research Ethics Committee of the Faculty of Dentistry, Universitas Islam Sultan Agung, Semarang (approval no. 603/B.1-KEPK/SA-FKG/IX/2024).

### Preparation of Nanoparticles from Saltwater Fish Powder

Nanoparticle saltwater fish powder was prepared from sardines ( *Sardinella fimbriata* ), splendid pony fish ( *Leiognathus splendens* ), and tuna ( *Euthynnus affinis* ). The fish were dried and ground into powder using an Ultra Turrax (IKA, Germany). Semi-quantitative XRF (SQX) analysis of the saltwater fish powder sample revealed that the dominant elements were calcium (Ca) at 8.50 mass% and phosphorus (P) at 3.68 mass%, with a calculated Ca/P ratio of approximately 2.31. Gas chromatography–flame ionization detector (GC-FID) analysis, using method code 18-6-1/MU/SMM-SIG, revealed that the saltwater fish powder nanoparticles contained 74.2 mg/100 mg of omega-3, 647.6 mg/100 mg of omega-6, and 16,675.5 mg/100 g of omega-9 fatty acids.

The powder was mixed with nanochitosan dissolved in 2% acetic acid and sodium tripolyphosphate, then homogenized using a magnetic stirrer (IKA, Germany). Particle size analyzer analysis using the dynamic light scattering method revealed a particle size distribution ranging from approximately 61 to >7,000 nm, with several main distribution peaks. The sample exhibited a nanoparticle fraction at approximately 91 nm, with a Z-average of 166.7 nm and an average of 273.3 nm, along with a particle size distribution extending into the micrometer range.

### Experimental Animals

Healthy mice ( *Mus musculus)* displaying normal behavior without lethargy or wounds were selected. A total of 16 female mice and 8 males (weighing 20–30 g, aged 2–3 months) were used. The mice were housed under controlled conditions (temperature: 23–27°C, light-dark cycle: 12 hours) with ad libitum access to food and a standard laboratory rodent diet (commercial pellet feed). Female mice's estrous cycles were monitored via vaginal swabs, and those in estrus were mated with males (ratio: four females to two males). A vaginal plug confirmed day 0 of gestation.

In addition to ad libitum access to a standard rodent pellet diet and water, the treatment group received oral administration of saltwater fish powder nanoparticles (2.14 mg/mL) dissolved in distilled water three times daily, while the control group received the same volume of distilled water (vehicle control) for 17 days.

### Sample Collection and Analysis

On gestational day 18, the mice were sedated with 10 to 20 mL of chloroform. Respiratory and heart rates were monitored before cervical dislocation. Placenta and fetal jaws were collected, fixed in 10% formalin, and processed for histological and immunohistochemical (IHC) analysis. The human protein atlas database was used as a reference for antibody selection and staining protocols to ensure specificity for murine tissues.

### Amelogenin and FABP-3 Expression Evaluation

IHC analysis was performed to evaluate the expression of amelogenin, a key marker of enamel matrix formation during odontogenesis in the developing tooth buds of fetal mice, and FABP-3 marker of transport and accumulation of fatty acids in placental tissues. Tissue sections were stained to visualize amelogenin and FABP-3 distribution in the tooth bud and placental tissues, respectively, and examined under a light microscope.

A mouse monoclonal antibody against amelogenin (F-11):sc-365284 (Santa Cruz Biotech Inc., Delaware, California, United States) was used as the primary antibody. A rabbit polyclonal FABP3 bs-11283R-HRP (Bioss ANTIBODIES, Boston, Massachusetts, United States) was used as the conjugated primary antibody. Amelogenin expression was measured 10 times per sample using a light microscope (Leica DM 750, Germany) at magnifications of 100× and 400×. Meanwhile, the FABP-3 analysis was conducted on 10 fields per sample of placental tissue.

### Enamel Density Evaluation

Enamel density was measured using µCT (SkyScan1173; Bruker, Kontich, Belgium). The µCT analysis was conducted by examining the average enamel density through sagittal, axial, and anteroposterior sections. Eight fetal mouse samples were used in both the control and treatment groups. The system used cone beam X-ray transmission with a flat-panel CCD camera (50 µm pixel size). The scanning parameters included a source voltage of 70 kV, tube current of 100 µA, image pixel size of 15 µm, exposure time of 650 ms, and a rotation step of 0.2 to over 360 degrees.

Image reconstruction was performed using NRecon software version 1.7.3.1 (Bruker; Kontich, Belgium) through Hamming-filtered back projection, with the following parameters: smoothing (1), smoothing kernel (2—Gaussian), and ring artifact correction (5). µCT images were resliced parallel to the axial plane, and the microarchitecture analysis was performed on consecutive slices or with multiplanar image reconstructions. The software rendered enamel structures in pink to facilitate identification.

### Data Analysis

Amelogenin and FABP-3 expression levels were quantified using ImageJ for IHC-stained sections and expressed in arbitrary units (AU). Tooth density was measured via micro-CT imaging and reported in Hounsfield units (HU), allowing comparison of mineralization between groups. All numerical data were analyzed using IBM SPSS Statistics 26. Data are presented as mean values ± SD. The data showed normal distribution and homogeneous variability ( *p* > 0.05); thus, the variable means between the control and treated groups were compared using the *t* -test. A *p* -value less than 0.05 ( *p* < 0.05) was considered statistically significant.

## Results

**1. Saltwater fish powder nanoparticle-enhanced amelogenin and FABP-3 expression**

The immunohistochemical staining in Figs 1 and 2 shows that the treatment group exhibits more intense staining, indicating higher protein expression. Specifically, Fig. 1 highlights pronounced amelogenin presence (red square) at 100× and 400× magnifications, suggesting enhanced enamel matrix formation. Similarly, Fig. 2 shows elevated FABP-3 expression, with more intense brown staining in the treatment group at both magnifications.

**Fig. 1:**
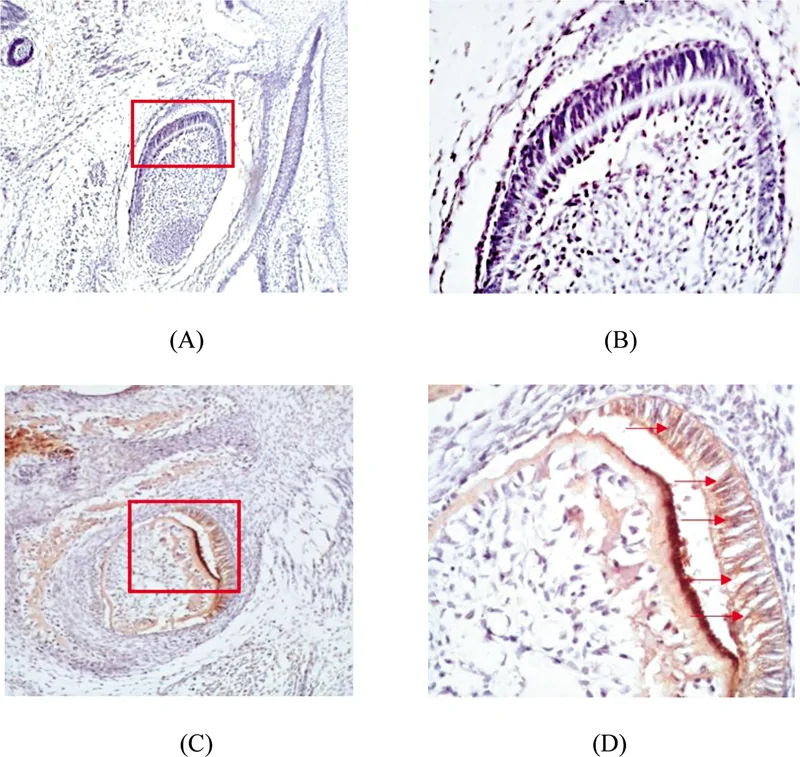
Amelogenin expression (highlighted in red squares and indicated by red arrows) in the tooth buds of fetal mice: ( **A** ) Control group at 100× and ( **B** ) 400× magnification; ( **C** ) treatment group receiving nanoparticle saltwater fish powder at 100× and ( **D** ) 400× magnification. The red arrows point to ameloblast cells exhibiting positive amelogenin expression.

**Fig. 2:**
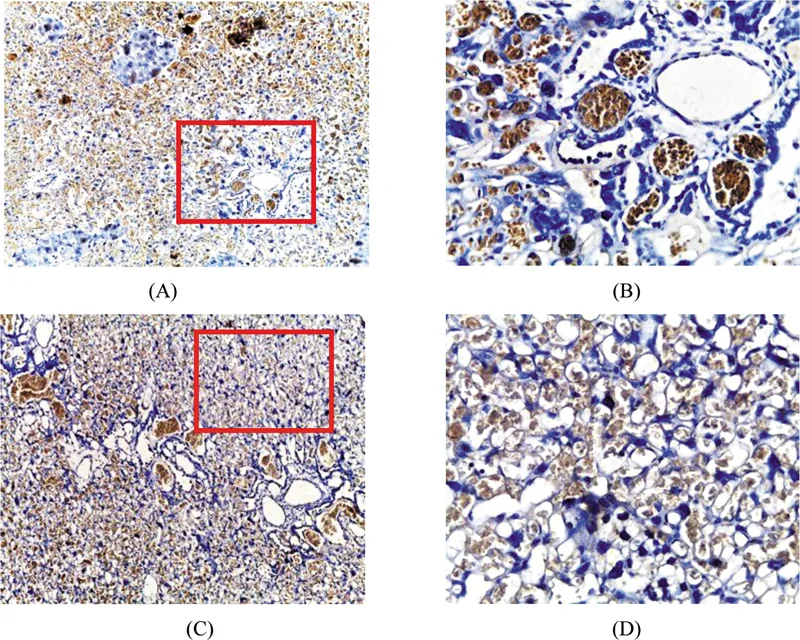
FABP-3 expression (red square) in fetal mice placental tissue: ( **A** ) control group at 100× and ( **B** ) 400× magnification; ( **C** ) treatment group receiving nanoparticle saltwater fish powder at 100× and ( **D** ) 400× magnification. More intense brown staining indicates higher FABP-3 expression in the treatment group.

The differences in staining intensity and color between the control and treatment groups are likely due to variations in the level of amelogenin protein expression. In immunohistochemical staining, higher protein expression typically results in darker or more intense staining, whereas lower expression produces lighter staining. These differences do not reflect inconsistent staining protocols, but somewhat true biological variation in amelogenin expression between the groups. All slides were stained under identical conditions to maintain consistency.

Quantitative analysis revealed significant differences between groups ( Table 1 ). The mean amelogenin expression, measured as the mean staining intensity using ImageJ analysis of IHC-stained sections, was 0.49 ± 0.40 AU in the control group and increased significantly to 4.34 ± 3.26 AU in the treatment group ( *p* = 0.005). FABP-3 expression was also significantly higher in the treatment group (2.26 ± 0.85 AU) than in the control (1.50 ± 0.40 AU), with a *p* -value of 0.038.

**Table 1:** The numerical data of amelogenin and FABP-3 expression in control and treated groups

Variables	Control group	Treated group	*p* -Value
Amelogenin expression (AU)	0.49 ± 0.40	4.34 ± 3.26	0.005 a
FABP-3 expression (AU)	1.50 ± 0.40	2.26 ± 0.85	0.038 a

a A significant difference ( *p* < 0.05) based on the *T* -independent test.

**2. Saltwater fish powder nanoparticle enhanced the enamel density in** μ **CT analysis**

Additionally, μCT imaging displayed structural differences in enamel density between groups. The enamel (pink) was more apparent and presumably denser in the treatment group ( Fig. 3B ) compared to the control ( Fig. 3A ), with the red arrows indicating the area of interest.

**Fig. 3:**
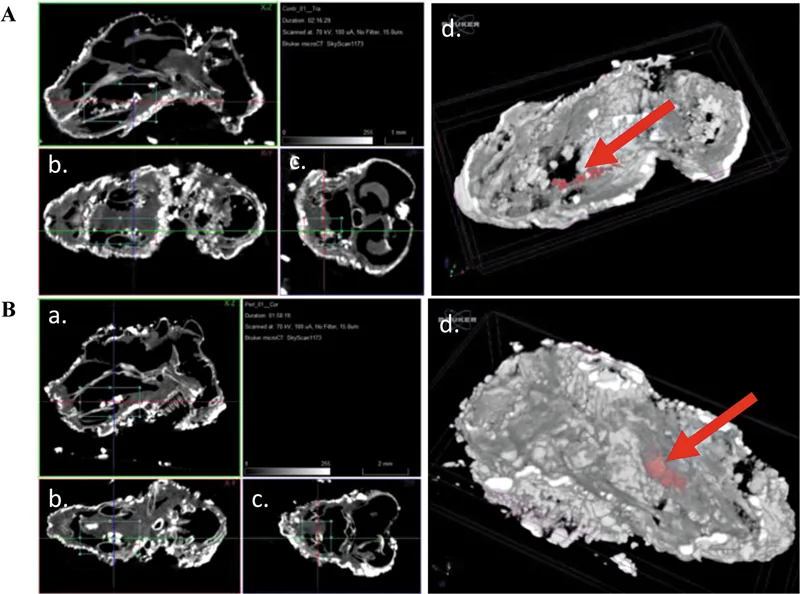
Micro-computed tomography (μCT) scan of a fetal mouse head. The red arrow indicates the region of interest (ROI), specifically the developing tooth germ area: ( **A** ) control group and ( **B** ) treated group—(a) sagittal section, (b) axial section, (c) anteroposterior section, and (d) 3D-reconstruction. The enamel is rendered in pink.

Quantitative analysis revealed a significant increase in enamel density in the treated fetal mice (228.73 ± 5.31 HU) compared to the controls (220.75 ± 5.95 HU), with a *p* -value of 0.022 (see Table 2 ).

**Table 2:** The numerical data of enamel density in control and treated groups

Variable	Control group	Treated group	*p* -Value
Enamel density (HU)	220.75 ± 5.95	228.73 ± 5.31	0.022 a

a A significant difference ( *p* < 0.05) based on the *T* -independent test.

## Discussion

This study demonstrates that the administration of nanoparticle saltwater fish powder significantly enhances amelogenin, FABP-3 expression, and enamel density during tooth development. These findings suggest nanoparticle formulations improve bioavailability and biological efficacy, positively influencing the key molecular pathways involved in amelogenesis. The observed improvements in both protein expression and enamel density underscore the therapeutic promise of this approach in supporting optimal dental tissue formation.

Amelogenesis is a highly coordinated process involving multiple distinct stages characterized by morphological and functional changes in ameloblasts. 3 17 The early presecretory stages involve odontoblasts-driven predentin, which initiates mineralization at the dentinoenamel junction. Following dentin mineralization, preameloblasts differentiate into secretory ameloblasts, which produce enamel matrix proteins, predominantly amelogenin. 3 17 18 Amelogenin plays a crucial role in regulating mineral deposition and controlling the morphology of enamel crystals. 6 7 19 In this study, immunohistochemical analysis revealed significantly increased amelogenin expression in the treatment group receiving nanoparticle saltwater fish powder (4.34 ± 3.26) compared to the control group (0.49 ± 0.40), with a *p* -value of 0.005. This upregulation indicates enhanced ameloblast activity and suggests that the bioactive components within the nanoparticle formulation could stimulate intracellular signaling pathways such as Wnt/β-catenin and BMP pathways, both of which are well-documented regulators of ameloblast differentiation and enamel biomineralization. 20 The improved amelogenin expression likely contributed to the increased enamel density observed via μCT.

Previous studies by Christiono et al using saltwater fish powder (non-nanoparticle) reported a mean amelogenin expression of approximately 3.13. 6 The higher amelogenin expression observed in this study (4.34 ± 3.26) suggests that nanoparticle delivery enhances the bioavailability of bioactive nutrients, facilitating a more pronounced stimulation of ameloblast activity and protein expression during the critical presecretory-to-secretory transition. This enhanced delivery aligns with findings from nanotechnology research, which indicate that reducing particle size to 50 to 300 nm improves cellular uptake and tissue penetration. 21

Calcium plays a crucial role in the mineralization of enamel and dentin. The protein calbindin-28kDa facilitates calcium diffusion and buffering within the ameloblast, influencing the formation of nanospheres and the crystallization of hydroxyapatite. 22 23 Calcium ions can induce conformational changes in amelogenin, thereby enhancing its self-assembly into nanospheres that regulate the growth of hydroxyapatite crystals. 24 The increased calcium content in the nanoparticle fish powder likely contributed to higher calcium bioavailability, supporting enamel mineralization. Recent studies have also highlighted innovative biomaterials that complement nutritional strategies in promoting enamel remineralization. Dhivya et al reported that strontium-doped fluorophosphate glasses exhibited enhanced ion release, improved bioactivity, and promoted apatite formation, thereby demonstrating potential for effective surface enamel repair. 25 In parallel, Saravana Karthikeyan et al demonstrated that both synthetic and eggshell-derived nanohydroxyapatite incorporated into carboxymethyl chitosan matrices facilitated biomimetic mineral deposition with favorable cytocompatibility, highlighting their applicability as sustainable and bioactive restorative agents. 26 Collectively, these findings suggest that ion-releasing glass systems and nanoparticle-based composites provide complementary mechanisms of action, which may be further optimized or integrated to achieve superior clinical outcomes in enamel remineralization.

Omega-3 fatty acids, including DHA, EPA, and alpha-linolenic acid (ALA), present in saltwater fish, are known for their anti-inflammatory and osteogenic effects. These fatty acids have been reported to promote calcium absorption, osteoblast differentiation, and lipid oxidation, collectively supporting mineralization processes. FABPs are a family of intracellular lipid-binding proteins that play a critical role in the uptake, transport, and metabolism of long-chain fatty acids. Among the different isoforms, FABP-3 (heart-type FABP) is particularly important in placental tissue due to its high affinity for polyunsaturated fatty acids, including DHA and EPA. Transport of omega-3 fatty acids to placental tissue is mediated by FABP-3. 27 Our observation of increased FABP-3 expression in the fetal trophoblast tissue indicates facilitated transport of omega-3 fatty acids across the placenta, enhancing fetal access to these nutrients. 28 By mediating the selective uptake and intracellular transport of these essential fatty acids, FABP-3 ensures adequate fetal supply.

The anti-inflammatory properties of omega-3s may also protect ameloblasts from oxidative stress, thereby maintaining their functional integrity during enamel formation. 6 Indahyani et al suggested that omega-3 supplementation affects tooth maturation and overall mineralization quality by modulating cytokines and growth factors involved in odontogenesis. 27 Similarly, Permatasari et al reported that omega-3 administration during pregnancy in mice enhanced the mineral composition of enamel by increasing CaO and P _2_ O _5_ levels. 29 Although direct human studies on enamel density are still limited, these findings collectively support the hypothesis that omega-3 fatty acids contribute to enamel development and mineralization.

Adding chitosan, a natural biopolymer derived from chitin, enhances membrane permeability and facilitates the penetration of nanoparticles across biological barriers, including the placenta. 16 Its properties, such as increased membrane contact and opening of tight junctions, allow for more efficient delivery of calcium, phosphorus, and omega-3 fatty acids to fetal tissues, including ameloblasts. Improved nutrient transport correlates with increased ionic availability, promoting mineralization and matrix organization, reflected in the higher enamel density observed via μCT analysis. 21 30

The findings of this study suggest that nanoparticle-based nutrient delivery systems hold significant potential for prenatal interventions to optimize dental enamel development. Such strategies could be particularly beneficial in populations vulnerable to mineral deficiencies or developmental enamel defects, such as amelogenesis imperfecta or fluorosis. Further research should investigate the detailed molecular signaling pathways involved, including those regulating Wnt/β-catenin, BMP, and other pathways critical to tooth morphogenesis. Longitudinal studies are warranted to investigate the durability and functional integrity of the newly formed enamel. Additionally, examining the effects of other bioactive compounds incorporated into nanoparticle formulations could expand the therapeutic spectrum. Translational research should also assess the safety profile and optimal dosing of nanoparticle nutrients to ensure efficacy without adverse effects. This study was conducted during the pre-secretory stage; therefore, the findings only reflect early amelogenin expression and may not fully represent its functional role in the later stages of enamel development.

## Conclusion

Nanoparticle saltwater fish powder demonstrates promising potential in enhancing amelogenin and FABP-3 expression, promoting mineralization, and increasing enamel density during fetal development. These findings underscore the importance of integrating nanotechnology with nutrient supplementation strategies to improve prenatal dental health outcomes.
